# The bone marrow aspirate and biopsy in the diagnosis of unsuspected nonhematologic malignancy: A clinical study of 19 cases

**DOI:** 10.1186/1471-2407-5-144

**Published:** 2005-11-01

**Authors:** Fahir Ozkalemkas, Rıdvan Ali, Vildan Ozkocaman, Tulay Ozcelik, Ulku Ozan, Hulya Ozturk, Ender Kurt, Turkkan Evrensel, Omer Yerci, Ahmet Tunali

**Affiliations:** 1 Division of Hematology, Department of Internal Medicine Uludag University School of Medicine, Uludag University Hospital, Bursa, Turkey; 2 Department of Pathology, Uludag University School of Medicine, Uludag University Hospital, Bursa, Turkey; 3 Division of Medical Oncology, Department of Internal Medicine Uludag University School of Medicine, Uludag University Hospital, Bursa, Turkey

## Abstract

**Background:**

Although bone marrow metastases can be found commonly in some malignant tumors, diagnosing a nonhematologic malignancy from marrow is not a usual event.

**Methods:**

To underscore the value of bone marrow aspiration and biopsy as a short cut in establishing a diagnosis for disseminated tumors, we reviewed 19 patients with nonhematologic malignancies who initially had diagnosis from bone marrow.

**Results:**

The main indications for bone marrow examination were microangiopathic hemolytic anemia (MAHA), leukoerythroblastosis (LEB) and unexplained cytopenias. Bone marrow aspiration was not diagnostic due to dry tap or inadequate material in 6 cases. Biopsy results were parallel to the cytological ones in all cases except one; however a meticulous second examination of the biopsy confirmed the cytologic diagnosis in this patient too. The most common histologic subtype was adenocarcinoma, and after all the clinical and laboratory evaluations, the primary focus was disclosed definitively in ten patients (5 stomach, 3 prostate, 1 lung, 1 muscle) and probably in four patients (3 gastrointestinal tract, 1 lung). All work up failed in five patients and these cases were classified as tumor of unknown origin (TUO).

**Conclusion:**

Our series showed that anemia, thrombocytopenia, elevated red cell distribution width (RDW) and hypoproteinemia formed a uniform tetrad in patients with disseminated tumors that were diagnosed via bone marrow examination. The prognosis of patients was very poor and survivals were only a few days or weeks (except for 4 patients whose survivals were longer). We concluded that MAHA, LEB and unexplained cytopenias are strong indicators of the necessity of bone marrow examination. Because of the very short survival of many patients, all investigational procedures should be judged in view of their rationality, and should be focused on treatable primary tumors.

## Background

Diagnosis and management of many hematologic diseases depends on examination of the bone marrow, which usually involves two separate specimens: a cytologic and a histologic preparation. While cytologic preparation of bone marrow, obtained by aspiration, allows excellent visualization of cell morphology, the second one, usually obtained with a Jamshidi needle, allows optimal evaluation of cellularity, fibrosis or infiltrative disease. In addition to hematologic malignancies, bone marrow examination has been increasingly useful in documenting metastatic involvement of tumors. During the past four decades, prospective evaluation of bone marrow aspirates and biopsy specimens has come into widespread use for accurate staging of many malignant diseases. Recognition of metastasis in random biopsies presents challenges to hematologists and pathologists when diagnosing the primary focus [1,2]

Although marrow metastases can be found commonly in some tumors, especially when newer sensitive methods are applied for the detection of tumor cells, diagnosing a nonhematologic malignancy from marrow is a rare event. We reviewed 19 patients with nonhematologic malignancies who were diagnosed initially from bone marrow, to underscore the value of bone marrow aspiration and biopsy as a short cut in establishing a diagnosis for disseminated tumors. Additionally we reported the details of the management and survival of the cases to offer a practical suggestion about work up of these patients to find a primary focus.

## Methods

This study is based on our retrospective analysis of 19 patients with solid tumors whose diagnoses were made from bone marrow seen at the Department of Hematology, Uludag University Medical Faculty, over a period of 9 years. Patients with non-Hodgkin's lymphomas and Hodgkin's diseases were not included in this study and patients with known neoplastic disease were also excluded.

The standard technique was employed in obtaining the samples from posterior iliac crest using a Jamshidi needle (Regular/Adult, 11-gauge). All of the trephine biopsies were performed unilaterally, because clinically none of the diseases that focally involve the bone marrow were included in the differential diagnosis prior to the biopsy procedure. Length of the biopsy cores ranged between 1.2 cm and 2.2 cm (mean 1.7 cm). Trephine biopsies were fixed in 10% neutral buffered formaline for at least 24 hours, and then decalcified overnight in a decalcifying solution which is a mixture of 8% HCl and 10% formic acid at equal amounts of volume. Following the automated tissue processing, biopsies were embedded in parafin blocks, and 0.3 micrometer sections were cut. In some cases the sternum was sampled using a Rosenthal needle for aspiration. Touch preparations were done if the aspiration resulted in a "dry tap" or if aspiration material was considered to be technically inadequate for evaluation, or if it was hemodilute. Bone marrow aspiration, touch preparations and peripheral blood smears which were obtained at the same time by biopsy were stained by May Grunwald-Giemsa. Hematoxylene-eosin, Giemsa and reticulin stains were routinely performed in biopsy sections. If the nonhematologic properties of the tumor could be identified with routine stains, and if the primary of the metastatic tumor could easily be diagnosed morphologically with routine hematoxylene-eosin stains, as in the cases of signet ring cell carcinoma; no immunohistochemical study was held. The pathologist's approach to definitive diagnosis of the patient with metastasis of unknown primary effectively followed a few sequential steps: First of all we tried to determine the cell line of differentiation e.g. carcinoma, lymphoma, melanoma, sarcoma, or germ cell, with the help of morphological findings and if needed, immunohistochemical stains. Our panel of antibodies contained pancytokeratin, HMB45, Leukocyte Common Antigen (LCA), Vimentin and Placental Alkalen Phosphatase (PLAP). If it was an epithelial tumor we tried to determine the cytokeratin (CK) type or types of distribution in the tumor cells, since some subsets of cytokeratins are uniqe to certain tumor types. Our panel contained AE1/AE3, CAM5.2, CK7, CK20, and 34 beta E12. In our further studies we tried to determine if there was expression of supplemental antigens of epithelial or germ cell derivation, that was Carcinoembryonic Antigen (CEA), Epithelial membrane Antigen (EMA) or PLAP. Last step was to determine if there was expression of cell-specific structures or receptors that are unique identifiers of cell types, for example neuroendocrine granules, peptide hormones, thyroglobulin, Prostate Specific Antigen (PSA), Gross Cystic Disease Fluid Protein (GCDFP) or Thyroid Transcription Factor-1 (TTF-1). Our panel of antibodies contained Synaptophysin, Chromogranin, Neuron Specific Enolase (NSE), Thyroglobuline, PSA, GCDFP, and TTF-1. Cases with sarcomatous properties were immunostained with Desmin, Smooth Muscle Actin, S100, Vimentin and Myoglobulin. After the confirmation of original peripheral blood and bone marrow cytological findings and histopathological diagnoses by a senior hematologist and an expert pathologist the patient charts were reviewed. Cases with a history of malignancy at the time of presentation were excluded.

Patient characteristics were recorded in each case, including: presenting symptoms, onset of symptoms, physical examination findings, peripheral blood counts, peripheral blood morphology, diagnostic evaluation, management, and survival.

## Results

Between 1995 and 2004, bone marrow metastasis was diagnosed in 19 samples among 5420 bone marrow aspirations and 856 bone marrow trephine biopsies. The ages of the patients were between 25 and 83 years (median age: 56), eight of them were female.

The main indications for bone marrow examination were microangiopathic hemolytic anemia (MAHA), leukoeryhtroblastosis (LEB) and peripheral cytopenias. Constitutional symptoms and pain were the most prominent presenting symptoms of the patients. Clinical findings were highly variable according to the underlying disease.

In the laboratory, the most common findings were anemia and thrombocytopenia, which were found in all patients. White blood cell counts (WBC) were between 3.3 × 10^9^/l and 17.1 × 10^9^/l (median: 8.1 × 10^9^/l), red cell distribution width (RDW) was increased in all cases, whereas mean corpuscular volume (MCV), mean corpuscular hemoglobin (MCH), reticulocyte percentage (RET) and erythrocyte sedimentation rate (ESR) were highly variable. Coagulation tests were carried out in 17 patients and in 8 of them at least one anomaly was detected at presentation; additionally, in two patients who had normal test results initially subsequent tests were found abnormal. All patients had hypoproteinemia; the second most common anomaly was elevated serum lactate dehydrogenase (LDH) level, which was found normal in only one patient (patient # 13) in biochemical analyses; some hepatic and renal parameters were abnormal in some patients but none of them was a constant finding.

In 6 cases bone marrow aspiration was not diagnostic due to dry tap or inadequate material. Histopathological examinations confirmed the nonlymphohematopoietic cell infiltration in first evaluation except one (patient # 15). However, a meticulous second evaluation of biopsy confirmed the cytologic diagnosis in this patient too. The most common histologic subtype was adenocarcinoma. After the all clinical and laboratory evaluations, the primary focus was disclosed in ten patients definitively (5 stomach, 3 prostate, 1 lung, 1 muscle) and in four patients probably (3 gastrointestinal tract, 1 lung). In five patients all work up failed and these cases were classified as tumor of unknown origin (TUO).

The prognosis of patients was very poor and survivals were only a few days or weeks except for 4 patients whose survivals were longer (patients # 9, 10, 17, 18). To avoid missing the individual features, detailed tables were prepared: Clinical and cytopathologic characteristics are shown in Table 1, clinical courses and survivals are shown in Table 2, and some hematological and biochemical features are shown in Table 3.

**Table 1:** Clinical and cytopathological characteristics of patients

No	Age/sex	Presenting symptoms/Onset of symptoms/Presence of constitutional symptoms	Performance status*/Physical findings	Cytological examination of peripheral blood	Cytological examination of bone marrow	Pathological examination of bone marrow
1	50/M	Back and chest pain/3 weeks/WL, F, NS	3/Pallor, subicterus	MAHA, LEB	Dilute, not optimal, not diagnostic	Adenocarcinoma with signet ring cell features (Suggestion: primary focus should be searched in GI tract)
2	40/M	Abdominal pain, failure to passage gas and stool by rectum, hematemesis/2 weeks/NS	3/Ecchymoses, tenderness in epigastrium and lower-right quadrant	MAHA, LEB	Dilute, not optimal, not diagnostic	Adenocarcinoma
3	41/F	Lumbar and extremity pain, lack of appetite, nausea/4 weeks/WL, F	3/Pain with deep palpation of whole abdomen	LEB, MAHA	Dilute, not optimal, not diagnostic	Adenocarcinoma
4	48/F	Lack of appetite, fatigue, nausea, vomiting, fever/2 months/F, WL	3/A few ecchymoses	LEB	Dry tap; touch preparation is not optimal for evaluation	Indifferentiated carcinoma (only CK positive strongly)
5	49/F	Dyspepsia, weakness/1 month/F	3/Pallor, multiple ecchymoses, axillary single microLAP	LEB	Dense foreign cell infiltration forming groups (adenocarcinoma)	Adenocarcinoma
6	71/M	Lumbar and leg pain, somnolence/20 days/-	4/Pallor, impaired consciousness, dysorientation, dyscooperation, agitation	LEB	Dry tap; imprint: highly dense atypical somewhat large round or oval cells infiltration in clusters	Atypical epithelial cells in clusters (round cells infiltration) (Suggestion: Primary focus should be investigated in lungs)
7	63/M	Cough, dysphagia, abdominal swelling, weakness, prominent loss in weight/WL, F, NS/1 month	2/Scleral icterus, 5 cm hepatomegaly, melana in rectal digital palpation	LEB	Epithelioid cells in solid clusters (small cell carcinoma infiltration)	Small cell carcinoma
8	57/F	Back, lumbar and leg pain, weakness, lack of appetite/3 months/WL	3/Scleral icter, left axillary 2 cm LAP	MAHA, LEB	Infiltration with signet ring cells	Metastatic carcinoma (signet ring cell adenocarcinoma)
9	45/M	Lumbar and leg pain, prominent loss in weight, generalized body pain/2 months/WL	3/Pallor;	LEB	Infiltration with atypical large epithelioid cells	Metastatic carcinoma (Suggestion: Primary tumor should be investigated in prostate)
10	25/F	Weakness, hip pain/6 months/ WL	2/Pallor, right inguinal 2 cm LAP, 1 cm hepatomegaly, 2 cm splenomegaly	Rare blastic cells	Infiltration with blastic cells with vacuolated cytoplasm (MPO negative; flow: B and T cell markers negative)	Higly dense atypical cells in alveolar structure-actin, desmin and vimentin positive, LCA and CK negative-(metastatic alveolar rhabdomyosarcoma)
11	35/M	Nausea, vomiting, prominent loss of weight, fatigue (alcohol, hashish and heroin dependence)/1 month/F, WL	4/Pallor, cachexia	MAHA	Adenocarcinoma cell forming groups	Metastatic adenocarcinoma
12	83/F	Prominent loss in weight, nausea, vomiting; backpain/6 months/WL	3/Pallor; multipl ecchymoses	MAHA, LEB	Infiltration with signet ring cell carcinoma cells	Metastatic adenocarcinoma (signet ring cell carcinoma metastasis)
13	61/F	Headache, sore throat, abdominal pain, constipation, nausea, vomiting (hematemesis and melana history), weakness/2 months/-	2/Pallor and scleral icterus, pain with palpation of right hypochondrium	LEB	Dry tap in first 2 attempts and very dilute without particle in 3^rd ^attempt. No atypical cells; imprint: technically inadequate	Metastatic carcinoma (CK positive atypical epithelioid cell infiltration, some of them in signet ring cell shape)
14	75/M	Multiple ecchymoses on body and on extremities, purpura on lower extremities; hematuria/2 days/-	1/Ecchymoses and purpura; 1.5 cm supraclavicular LAP	Only minimal shift to left; no erythroblast or poikilocytosis	Non-hematopoetic cell infiltration forming groups and some with mucineous character	Metastatic adenocarcinoma (CK+ cells forming glandular and tubular structures
15	73/M	Confusion, adynamia/20 days/WL	4/Hypotension, hypothermia, dehydration, scleral icterus, pallor, cachexia, 2 cm hepatomegaly	Slight shift to left, toxic granulation, slight poikilocytosis, no erythroblast, no fragmentation	Dilute and not optimal but there are nonhematopoietic cells in small groups like adenocarcinoma cells	Nondiagnostic in first report but adenocarcinoma metastasis reported after meticulous examination of new further sections of blocks
16	75/F	Dyspnea, abdominal swelling/20 days/-	4/Rales bilaterally, 2 cm hepatomegaly, pretibial edema, petechia in lower extremities	LEB, no poikilositosis or fragmentation	Atypical non-haematopoetic cells (adenocarcinoma metastasis)	ND
17	68/M	Weakness, lack of appetite, prominent loss of weight, lumbar pain/3 months/WL	1/Enlargement and nodulation in prostate in digital examination	LEB, slight poikilocytosis, no fragmentation	Infiltration with adenocarcinoma cells showing acinar and tubular structures	Metastatic carcinoma compatible with prostate carcinoma (PSA +)
18	56/M	Pain in hips and legs, weakness/2 months/F, WL, SN	2/Pallor, cachexia, multiple microLAPs in servical, axillary and inguinal regions, a few petechiae and eccyhymoses	LEB, MAHA	Infiltration with atypical epithelioid cells forming papillar and acinar structures (adenocarcinoma metastasis)	CK + PAS- adenocarcinoma metastasis (Suggestion: Primary focus should be investigated in prostate)
19	45/M	Neck and hip pain, abdominal pain, weakness/ 1 month/WL	3/restriction in physical activity	LEB, MAHA	Infiltration with adenocarcinoma cells	Metastatic adenocarcinoma; CK+ epithelioid cells, some of them mucinous and in shape of signet cell (Suggestion: Primary focus should be investigated in stomach)

*According to WHO/ECOG; WL: weight loss; F: fever; NS: night sweats; LAP: lymphadenopathy; MAHA: microangiopathic hemolytic anemia; LEB: leukoerythroblastosis; MPO: myeloperoxidase; CK: Cytokeratin LCA: leukocyte common antigen; ND: not done

**Table 2:** Evaluation, clinical course and survival of patients

	Evaluation for primary focus	Final decision for primary focus	Clinical course/Management	Survival
1	Chest XR, abdominopelvic USG and transrectal USG: N; CEA and CA15.3 are high slightly and Ca19.9 is high more than 10 folds, among CEA, α FTP, PAP, CA-125, CA19.9, CA15.3	GI tractus	General condition deteriorated rapidly; stupor and coma developed/Supportive care; 2 courses of TPE	6 days
2	Chest XR, abdominopelvic USG and CT: N; gastroscopy: malign ulcus	Stomach	DIC, subdural hematoma developed/Multiple erythrocyte, platelet and plasma transfusions; 2 courses of TPE	11 days
3	Abdominopelvic USG and CT: thickness on the antrum wall, gastrohepatic and portahepatic microLAPs; only α-FTP is in normal limits, among the CEA, α-FTP, CA 125, CA15.5; CA19.9 is high more than 10 folds; gastroscopy: infiltration in corpus and antrum (linitis plastica)	Stomach	DIC diagnosed at admission. Hematemesis and epistaxis developed later/Despite full transfusion support; died due to intracerebral bleeding	20 days
4	Chest XR, mammography: N; abdominopelvic USG and CT: N except minimal free pelvic fluid; upper GI tract endoscopy: unremarkable	TUO	Fever resisted despite AB; general condition deteriorated gradually, generalized seizures developed without abnormal cranial CT finding; hypoxemia developed due to secretions/Supportive care only	12 days
5	Chest XR: N; abdominopelvic CT: not optimal; suspected thickness on the stomach wall, suspected metastatic lesions in columna vertabralis; upper GI tract endoscopy: malign ulcus in cardia; CA125 is high slightly and CA-19-9 is high approximately 6 times, among CEA, α-FTP, Ca 125, CA 19-9, CA 15-3	Stomach	AB resistant fever (FUO) and GI tract bleeding developed later/Despite full transfusion support her general condition deteriorated rapidly. Died in MODS picture	37 days
6	Cranial CT: N; thorax, abdomen and pelvis MR: multiple mediastinal LAPs in conglemeration with suspected parenchimal infiltration, benign prostate hyperthrophy; lumbar MR: multiple pathologic signal in backbone and degenerative alterations; Skeleton scintigraphy: multiple thoracal and lumbar uptake (degeneration, metastasis, trauma?)	Lung?	His consciousness impaired progressively; refractory fever and hypotension developed	5 days
7	Thorax, abdominal CT: subcarinal LAPs, a mass in right hilus, eosophagial compression, pulmonary artery and pericardium invasion, a hipodens lesion in 1 cm diameter in liver (USG in terminal period: multiple lesions compatible with metastasis); bronchoscopy: inoperable bronchial carcinoma; biopsy: small cell carcinoma); upper GI tract endoscopy: N. CEA, α-FTP, PSA, freePSA, CA125, CA 19.9 all: N	Lung	Pneumonia and atrial fibrillation developed/A course of CT (Etoposide+Cisplatine) was given. Died duo to CHF	47 days
8	Axillary node FNAB: benign; cranial MR: compatible with bone metastasis and leptomeningeal carcinomatosis; thorax CT: only bone metastasis; abdominopelvic CT: 3 mm hipodens lesion in liver (metastasis?), backbone metastasis; transvaginal USG: N; bone scintigraphy: multiple metastasis; mammography: N; whole spine MR: generalized sclerotic and lytic lesions; upper GI tract endoscopy: erythemateous gastritis; only CA 125 is high 2 folds, among CEA, α-FTP, CA 125, CA 19-9, CA15-3, BHCG	TUO	Performance status deteriorated gradually. GI tract bleeding developed/She refused colonoscopy and other supportive therapies and was discharged in very bad condition	38+ days
9	Chest XR: N; transrectal USG: prostate carcinoma?; prostate biopsy: adenocarcinoma; skelatal XR survey: multiple sclerotic metastasis and compression fracture in L3; bone scintigraphy: generalized metastatic involvement; tumor markers: PSA and free PSA are very high	Prostate	After his work up bisphosphonates therapy was initiated and was fallowed as outpatient; cranial metastasis developed later; despite progressive complaints he refused admission	7+ months
10	Abdominopelvic CT: a solid mass in the pelvis originated probably right gluteal muscle and homogeneous hepatosplenomegaly; biopsy from the mass: rhabdomyosarcoma; skelatal XR survey: lytic lesions in only pelvic bones and proximal femur; bone scintigraphy: pathologic uptake in bilateral knee, pelvic area and, 5. and 7. ribs	Muscle	VAC/IE (Vincristine, Adriamycine, Cyclophosphomide, Ifosfamide, etoposide) therapy resulted in partial response; died because of progression later	7 months
11	Chest XR: N; abdominopelvic US: N except homogen minimal hepatomegaly	GI system?	His general condition deteriorated rapidly; GI tract bleeding and subdural hematoma developed, Died because of herniation/Supportive care only	10 days
12	Abdominopelvic CT: normal except suspected rigidity in stomach wall; gastroscopy: malign ulcus in junction of corpus and fundus; biopsy: Adenocarcinoma; Mammography: N; Backbone XR: loss of height in Th11 and Th12	Stomach	One course 5FU+FA was given; died as out patient	1 month
13	Nasopharynx biopsy: N; pleural fluid cytology: negative, biopsy: nonspecific chronic pleuritis; mammography:N; bone scintigraphy: multiple uptake; colonoscopy: N; gastroscopy: N; CEA and CA15.3: N, CA19.9 and CA125: very high	GI system	Transfusion support. Lost to follow up	45+ days
14	Chest XR and abdominopelvic USG: N	TUO	Nothing. Out of follow in 2^nd ^week	14 days
15	Chest XR: nondiagnostic; CEA, α-FTP, PSA, free PSA, CA15.3, Ca19.9, Ca125 all: N	TUO	Despite vigorous transfusion support and antibiotics his vital functions deteriorated progressively and died in MODS	4 days
16	Chest XR: nondiagnostic; previous available tests: thorax CT: linear atelectasis and minimal right pleural fluid, 1–2 cm multiple mediastinal LAPs;.abdominal CT: homogeneous hepatomegaly and multiple cysts in 1.5 cm diameter in head of pancreas	TUO	She died because of hypertensive crises and CHF after admission	1 day
17	Pelvic and transrectal USG: Prostatic hypertrophy; prostate biopsy: Adenocarcinoma; thoracolumbar MR and bone scintigraphy: multiple bone metastasis in backbone	Prostate	Flutamide (antiandrogen) and Goserelin asetat (LH-LR analogue) were given. Paraparesis and paraplegia unresponsive to RT developed and died because of progressive disease and CHF	15 months
18	Neck and thorax CT: N; abdominopelvic CT: paraaortic 1.5 cm LAPs and heterogen prostatic hypertrophy, prostate biopsy: Adenocarcinoma; pelvis XR: multiple sclerotic lesions; bone scintigraphy; multiple + focuses in whole skeleton: PAP and PAS: very high; CEA, AFP, CA19.9: N	Prostate	Gaserolin asetat+ Bikalutamid (LH-RH analogues) were given; (he was in a good condition when writing)	3+ months
19	Bone scintigraphy: multiple + uptake; gastroscopy: malign ulcus; biopsy: signet cell carcinoma (antrum); CEA, CA 19.9: very high, Ca125: high, AFP, PSA, F-PSA:N; thoracal MR: loss of height in Th8; toraks CT: frosted glass appearance in lower and middle zones, minimal pleural effusion bilaterally; abdominopelvic CT:N; skelatal XR: Lumbar and pelvic sclerotic lesions	Stomach	Supportive care and palliative RT were given; he died because of progressive disease	51 days

XR: direct radiography; CEA: carcinoembryonic antigen; α FTP: alpha fetoprotein; PAP: prostate specific antigen; BHCG: Beta human chorionic gonodotropin; USG: ultrasonography; CT: computed tomography; MR: magnetic resonance; Th: thoracal; LAP: lymphadenopathy; GI: gastrointestinal; DIC: disseminated intravascular coagulation; N: normal; FNAB: fine needle aspiration biopsy; TUO: tumor of unknown origin; TPE: therapeutic plasma exchange; AB: antibiotic; FUO: Febris of unknown origin; MODS: multiple organ deficiency syndrome; CHF: congestive hearth failure; RT: radiotherapy;

**Table 3:** Hematologic parameters of peripheral blood and biochemistry of serum of patients*

No	WBC (× 10^9^/l)	Hb (mg/dl)	MCV (fl)	MCH (pg)	RDW	PLT (× 10^9^/l)	RET (%)	ESR (mm/1 h)	Coagulation tests	Total Protein/Albumin	LDH	Total/Direct Bil	AST	ALT	ALP	Urea	Creatinine
1	8.1	9.9	94.5	33.3	17.0	32	2.5	17	PT and FDP: high	6.3/3.4	508	3.1/1.4	49	38	525	70	0.8
2	6.8	7.0	93.2	28.7	15.6	19	3.0	20	Ne initially and all abnormal later	5.6/3.0	1042	1.5/0.5	157	33	365	56	1.0
3	11.6	6.4	79.7	27.0	16.1	20	1.0	36	PT aPTT and FDP: abnormal	5.0/2.1	776	2.9/1.1	67	57	587	46	0.4
4	13.1	6.8	74.1	25.2	18.4	41	0.8	78	N	5.7/2.6	1669	0.9/0.5	104	48	282	148	2.1
5	15.8	6.6	82.0	26.3	16.7	18	3.5	34	N initially but abnormal later	5.8/2.7	1016	2.4/1.1	61	11	661	84	0.8
6	10.6	10.6	73.8	24.4	15.3	12	0.2	107	N	6.2/2.8	1255	0.5/0.3	52	22	240	89	1.2
7	4.2	9.3	85.7	29.2	15.2	21	1.4	80	N	5.8/3.6	1438	1.1/0.5	78	54	155	68	1.0
8	6.5	8.1	89.3	30.9	16.5	72	4.7	44	N	5.7/2.9	880	2.3/0.9	30	17	1548	33	0.5
9	6.4	9.5	61.9	20.5	16.3	45	1.0	65	N	6.4/3.4	665	0.5/0.4	41	14	433	43	0.3
10	7.7	5.3	79.3	27.5	15.8	11	0.0	140	N	5.7/2.2	1612	0.5/0.2	26	4	71	15	0.6
11	10.3	4.0	90.0	29.9	16.8	32	1.8	5	D-Dimer and PT: high	6.6/4.0	1899	1.3/0.7	78	22	262	48	0.8
12	11.5	7.0	84.7	26.4	17.0	37	3.2	30	PT, aPTT and D-Dimer: high	6.7/4.6	904	1.8/0.7	130	78	402	63	1.2
13	6.5	6.4	90.7	28.6	17.3	103	2.8	40	N	6.6/3.5	258	4.4/1.4	11	10	1097	26	0.5
14	9.1	12.6	87.9	30.0	14.6	48	0.8	10	ND	6.2/3.0	630	1.1/0.4	41	25	144	29	0.9
15	3.3	3.3	89.3	30.2	17.7	24	0.4	95	PT, aPTT and D-Dimer: high	5.4/2.6	777	1.8/1.6	43	18	82	161	1.6
16	16.2	8.7	92.1	30.3	17.8	23	ND	85	ND	5.9/2.3	708	3.3/1.6	41	18	156	114	2.3
17	37.1	6.6	80.5	26.2	21.7	76	3.6	94	PT and D-Dimer high, aPTT: N	7.3/3.3	1127	1.2/0.5	70	48	1225	23	1.0
18	37.4	8.5	91.8	28.9	16.0	18	2.2	30	PT, aPTT and D-Dimer: high	6.1/3.9	393	1.7/0.6	27	36	1507	17	0.5
19	17.1	8.4	91.0	31.5	16.9	54	4.2	47	PT high D-Dimer upper limit, aPTT:N	6.2/3.8	1290	0.7/0.3	68	75	638	37	0.5

*Normal ranges of serum chemical parameters: Total protein: 6.4–8.8 g/dl; Albumin3.0–5.5 g/dl; LDH: 190–380 U/l; Total bilirubin: 0.2–1.1 mg/dl; Direct bilirubin:0.0–0.4 mg/dl; AST: 0–40 U/L; ALT: 0–43 U/L; ALP: 27–147 U/L; Urea: 15–50 mg/dl; Creatinine: 0.5–1.6 mg/dl; PT: prothrombin time; aPTT: active partial thromboplastin time; FDP: fibrin degradation products ND: not done; N: normal

## Discussion

Although bone marrow metastases are frequently encountered in patients with disseminated solid tumors, it is not a common event as a presenting sign. It stems from two possible reasons: i) in general, bone marrow is seldom the sole site of systemic involvement by malignant disease and ii) bone marrow examination in evaluating patients with suspected malignancy has a limited value unless supported by some other clinical finding, such as a leukoerythroblastic reaction [1,3,4]. Although there are a number of correlates that may be useful, there is no single specific finding that is an indicator of marrow infiltration. Furthermore, in most patients with neoplastic infiltration of the marrow, the peripheral blood findings do not differ significantly from those without marrow involvement. Our series showed that MAHA, LEB and unexplained cytopenias are strong indicators of the necessity of bone marrow examination. When we reviewed routine hematologic and biochemical parameters we found that only four were present in all patients: anemia, thrombocytopenia, elevated RDW and hypoproteinemia. However, our results should be interpreted with caution, as the current study was not designed to determine the predictive parameters of marrow metastases.

LEB is the term used to describe the combination of nucleated red cells (erythroblasts) and immature myeloid precursors (e.g. myelocytes and myeloblasts) in the peripheral blood film. The mechanism of leukoerythroblastic reactions is not defined. The invasion of metastatic cancer cells may cause the early release of some cytokines, leading to the development of a myelophthisic blood picture even before the marrow is completely replaced. This is a possible explanation for some instances of leukoerythroblastic changes in the blood of the patients with tumors in whom marrow metastases are not documented by histologic examination. Although metastatic foci can be found in a high percentage of some carcinomas, the development of frank LEB occurs much less frequently, so its absence should not exclude marrow involvement. If there is LEB in a case of suspected malignancy, bone marrow examination should be considered. Our series confirmed this judgment, because fifteen of the patients were presented with LEB. On the other hand despite clear bone marrow involvement in 4 patients there were no erythroid and myeloid precursors in their peripheral blood films (patients # 10, 11, 14, 15). LEB was reported at different rates in different series consisting of cancer patients with bone marrow metastases: 10/27 [5]; 19/25 [6]; 5/63 [7]; 26/73 [2]. Our relatively high LEB ratio (15/19) is not comparable with other series because they consisted of cancer patients who were diagnosed before bone marrow examination. Our high LEB ratio, when considered along with the bad outcomes, may reflect the late and advanced cases. In recent years there is a scarcity of publications on the association of myelophthisis with cancer. As mentioned by the authors in a recent report [8], early diagnosis and more effective therapies are possible explanations for this decrease.

MAHA or thrombotic microangiopathy (TM) describes the association of hemolytic anemia with red cell fragmentation caused by microangiopathy mechanically. Cancer related thrombotic microangiopathy (CR-TM) is a rare and severe complication; it usually occurs in the late or terminal stage of cancer with a short-term life-threatening prognosis [9,10]. CR-TM shares certain clinical similarities with thrombotic thrombocytopenic purpura such as neurological and renal impairment; also both are characterized by circulating platelet aggregates containing ultra large multimers of Von Willebrand factor (VWF). A recent report showed no VWF cleaving protease deficiency in a patient with metastatic adenocarcinoma of the colon and microangiopathic hemolysis, who is refractory to plasma exchange [11]. We do not have any data about protease activity but, because of an almost identical clinical picture with TTP as presentation, we started plasma exchange immediately in patient # 1 and # 2 but therapy failed as expected. Hemolysis, in our CR-TM patients, was so severe that several units of transfusion per day were required to maintain a safe hemoglobin level. Reticulocytosis is expected but this finding could not be a reliable indicator of hemolysis, as seen in our patients, because of marrow infiltration or chronic disease. In our series, seven out of eight MAHA patients showed LEB and six showed abnormality in some coagulation tests suggesting disseminated intravascular coagulation. When we reviewed our eight MAHA cases there were two TUO and one prostate carcinoma. All definitive stomach carcinomas and a probable gastrointestinal carcinoma were in the MAHA group. The survivals of our MAHA patients were between 11 and 51 days except for one (patient # 18; prostate carcinoma). This suggests that once microangiopathic hemolysis is seen in a patient with disseminated carcinoma, the overall prognosis is poor, especially in stomach adenocarcinoma presented with MAHA.

"Dry tap" is a term used to describe failure to obtain bone marrow on attempted marrow aspirations. Extensive marrow fibrosis and hypercellularity have been proposed as mechanisms to account for the inability to withdraw marrow by aspiration [12,13]. Because it can be attributed to faulty technique it should only be used retrospectively after review of the biopsy. We have two dry taps (patients # : 4, 6). If the definition of dry tap includes cases in which material is obtained but no, or inadequate marrow cells found in films we have four additional cases (patients # 1, 2, 3, 13). We concluded that bone marrow biopsy is certainly indicated whenever aspiration results in an insufficient material especially in the presence of LEB, MAHA, cytopenias and an elevated serum LDH.

Classically, marrow biopsy is unequivocally the best method of detecting lymphomatous involvement because in approximately one-third of cases, the aspirate is unremarkable and the biopsy shows tumor. In solid tumors other than those of lymphomatous origin the data in the literature comparing the relative value of bone marrow aspiration and biopsy in detecting marrow involvement are not so conclusive. [2,14]. Naturally trephine biopsies have a definitive advantage over aspirates in cases of dry tap. Additionally histologic sections may allow classification of the type of tumor cells, and this is of particular value in the investigation of a patient with a malignancy of unknown primary site. Aspirations were diagnostic in 12 patients in our series. The other attempts yielded no, or inadequate material. Cytological diagnoses could not be confirmed in one patient because of the patient's objection to biopsy procedure. Only in one instance was cytology superior to biopsy in first examination (a retrospective examination of biopsy confirmed the cytological finding in this patient). Although biopsies have some advantages, tumor cells occasionally can be seen in aspirate preparations when biopsy sections are normal as mentioned in literature, these two procedures should therefore be regarded as complementary.

The management of a patient, whose solid malignancy is disclosed from bone marrow, depends on his/her primary tumor. The pathologist can assist the clinician by thoroughly examining the histologic specimen. In some cases an immunoperoxidase staining for organ-specific antigen examination might be sufficient to bring out of the primary focus. As an example, an immunoperoxidase stain for prostatic acid phosphatase or prostate-specific antigen may be helpful in establishing a diagnosis of metastatic prostate cancer. On the opposite side of the spectrum in some cases the pathologist could not comment on the primary site because of a poorly differentiated tumor. Maximum effort should be made to minimize the target. In these tumors as a first step leukocyte common antigen may be used to differentiate lymphohematopoietic neoplasm from other cancers. Patient # 7 and 10, who were reported recently elsewhere separately [15,16], are good examples for bringing out the primary focus by extra stainings. The examination of patients with bone marrow metastases of unknown origin should focus on detecting treatable primary tumors [17]. This work up may result in certain improvements in survivals of patients with prostate carcinoma. Bone marrow metastases commonly arise from lung, breast and prostate cancers; therefore, in case of no clinical sign a reasonable work up should include a chest roentgenogram, a prostate examination with serum prostate specific acid phosphotase (PAP) in men; a breast examination and mammography in women; computed tomography scans also should be performed in suspected cases. Serum tumor markers are not so useful in many cases except PAP; considering their less specificity it is no surprising. Because the expected survival of many patients is quite short, laboratory and imaging studies should be considered in view of their rationality.

## Conclusion

In conclusion, MAHA, LEB and unexplained cytopenias are strong indicators of the necessity of bone marrow examination. Anemia, thrombocytopenia, elevated RDW and hypoproteinemia form a uniform tetrad in patients with disseminated tumors that are diagnosed via bone marrow aspiration and biopsy. Because of the very short survival of many patients, all investigational procedures should be focused on treatable primary tumors.

## Competing interests

The author(s) declare that they have no competing interests.

## Authors' contributions

FO, RA and VO participated in the conception and design of study, acquisition of data, analysis and interpretation of data. TO, UO, HO and EK participated in acquisition of data and drafting the article. TE, OY and AT participated in revising it critically for important intellectual content. All authors read and approved the final manuscript.

## Pre-publication history

The pre-publication history for this paper can be accessed here:


